# Effect of Additives and Print Orientation on the Properties of Laser Sintering-Printed Polyamide 12 Components

**DOI:** 10.3390/polym14061172

**Published:** 2022-03-15

**Authors:** Lucie Zárybnická, Jana Petrů, Pavel Krpec, Marek Pagáč

**Affiliations:** 1 Department of Technical Studies, College of Polytechnics Jihlava, Tolstého 16, 586 01 Jihlava, Czech Republic; lucie.zarybnicka@vspj.cz; 2 Department of Machining, Assembly and Engineering Technology, Faculty of Mechanical Engineering, VSB-TU Ostrava, 17. Listopadu 2172/15, 708 00 Ostrava, Czech Republic; jana.petru@vsb.cz; 3 V-NASS, A.S., Halasova 2938/1a, 703 00 Ostrava, Czech Republic; pavel.krpec@v-nass.cz

**Keywords:** powder bed fusion, polyamide 12, additives, print orientation, mechanical properties

## Abstract

3D printing, also known as additive manufacturing, is becoming increasingly popular for prototype processing in industrial practice. Laser sintering, which is a laser powder bed fusion technique, is a versatile and common 3D printing technology, which enables compact and high-quality products. Polyamide 12, a popular 3D printing material, provides reliable mechanical and thermal properties. Weaknesses in applying this technology for polyamide 12 include incomplete information regarding the application of various types of additives and different printing orientations with respect to the properties. This study aimed to investigate the influence of various additives (including carbon fiber, glass fiber, flame retardant, and aluminum powder) combined with polyamide 12, using processing of predefined powder refreshing mixture on the properties of a finished product. The thermal, surface, and mechanical properties of samples printed with five different polyamides 12-based powders at three different print orientations were investigated. It was found that the inclusion of additives decreases the tensile strength and increases the surface roughness of printed components—however, the toughness increases. The results can assist designers in selecting an appropriate material that will produce a finished part with the required properties for a given application.

## 1. Introduction

3D printing (3DP), also known as additive manufacturing (AM), rapid prototyping (RP), and solid-freeform (SFF), is a useful technology that can make objects from 3D model data. The first 3DP technique, vat photopolymerization (VPP), was first described in 1986 by Charles Hull [1]. Laser powder bed fusion (LPBF) is a technique where objects are made by selectively fusing a thin layer of polymer powder using a laser beam. The 3D object is then built up layer-by-layer [2,3]. LPBF is a good method for cost-effectively making components with complex geometrical features relatively quickly when compared to conventional manufacturing methods.

Thermoplastics, including polyethylene [4,5,6], polypropylene [7,8,9], and thermoplastic elastomer [10,11,12,13], are some of the most common groups of materials that are processed using LPBF technology. Technical thermoplastics based on various types of polyamide (PA) have great potential for the production of highly stressed components in various areas of application. They have high mechanical strength under both static and dynamic stress and are able to withstand long-term loading in a wide range of temperatures [14,15].

Many researchers have carried out research on pure PA 12 powder samples to determine their optical and thermal characteristics (thermal diffusion, thermal conductivity, influence of heating rate on shrinkage, etc.), molecular weight, and powder particle size and its influence on print quality. Studies have also been carried out comparing PA 12 3D printed and pressed objects [9,16,17,18,19,20,21,22,23,24,25,26,27,28]. Studies investigating composite materials, such as a PA 12-based polymer blended with tungsten carbide and cobalt (WCCo), are also available [14,29,30,31,32]. One study found that vacuum sintering and sinter-hot isostatic pressing (HIP) this polymer blend lead to a density of up to 66%, with shrinkage ranging from 36 to 40% [32].

Few studies addressing 3D print orientation and the inclusion of additives have been published. As part of their research, Tomanik et al. determined tensile and compressive tests for evaluating porosity [3]. Interestingly, the samples were not produced on an industrial 3D printer but on a desktop device. Similar to this study, Gua et al. investigated the surface thermal properties and flammability of a PA 12 composite material blended with glass fibers [33]. The samples were made using LPBF technology developed by Hewlett Packard.

A comparison of LPBF technologies using two different PA 12-based powders, EOS PA2200 and HP 3D HR, was made by Caia et al. [34]. In the study, 12 parts were compared, and their physicochemical characteristics and mechanical properties were evaluated. Both powders used had almost identical thermal properties, phase structure, and chemical composition. The mechanical strength of the samples produced by Hewlett Packard technology was slightly higher.

A study to determine the failure criterion for materials with anisotropic properties with stress interactions to predict the failure of Hewlett Packard components made using PA 12 powder was carried out by Osswald et al. [22]. Salmoria et al. studied the mechanical properties of pure PA 6, PA 12, and PA 6/PA 12 mixtures [35]. The paper dealt with laser sintering of powder material with a mixture ratio of 80/20, 50/50, and 20/80. They found that the blended powders had significantly lower strength than the pure powders due to the low affinity between the polar polymers PA 6 and PA 12. The results are important for the application, use, and development of new materials for LPBF technologies.

Fracture toughness of single edge notch bending samples under a three-point bending fixture, according to the ASTM D5045-14, was studied in [36] for PA_2200 and Alumide. The structural differences between PA_2200 and Alumide highly affected the fracture toughness, with the Alumide possessing KIC values of about 62–65% lower (orientation dependent) than those of PA_2200.

In the study [37], they monitored the effect of laser power on the properties of PA2200 material; they also monitored the positive effect of paraxylene solution on surface quality.

The structure and morphology of parts made from PA 12 material and produced by LPBF technology were studied by Martynkov et al. [38]. The subject of the research was the evaluation of crystallography using X-ray powder diffraction (XRPD), Fourier transform infrared spectroscopy (FT-IR), and differential scanning calorimetry (DSC) of the surface properties. One of the research goals was to compare the new and recycled powder in the powder bed. The results show that the degradation of the material depends on the used laser energy, and therefore the sintering of the powder material after recycling is less efficient.

It is clear that a great deal of research has been carried out with respect to the use of PA 12 with LPBF technologies. Combining PA 6 or PA 12 with various additives can increase material properties and enable wider application in the automotive, aerospace, and mechanical engineering industries. Popular additives include aluminum powder [39]; glass [40,41,42,43], and carbon fibers [44,45,46,47,48,49], as well as self-extinguishing particles (flame retardant) [50,51]; antistatic particles (electrostatic discharge) [52], and black carbon [53].

This paper summarizes the physicochemical and mechanical properties of these materials in terms of using different types of additives. The research findings provide a comprehensive understanding of the pure PA 12 powder (sample identification PA_2200) and other composite mixtures based on PA 12 with different additives such as flame retardant (sample identification PA_2210_FR), glass fiber (sample identification PA_3200_GF), aluminum powder (sample identification PA_Alumide), and a mix of glass and carbon fibers (sample identification PA_640_GSL). These variations of PA 12 combination with additives result in enhanced heat resistance and flame resistance, chemical resistance, and mechanical properties.

For individual types of powder mixtures, it is also important to mention before their processing the so-called refreshment factor. The refreshment factor is dependent on several factors. Firstly, the duration of the action of heat on the powder is important because the plastic powder is damaged more during a long building time and as the level to which the job is filled with parts, the more densely a job is filled with parts, the greater the energy applied by the laser, due to the greater exposure volume. As a consequence, the temperature in the exchangeable frame increases, and the plastic powder is damaged more. The values given specify the minimal refreshing with new powder and, in practice, may vary depending on the application and environmental conditions. Dimensional analysis of printed parts is also difficult to find in the literature. The information can be a guide for designers who wish to design their components using knowledge of product properties in different print orientations.

## 2. Materials and Methods

### 2.1. Materials

Five types of commercial PA 12 powders with and without additives were tested. These powders are marketed for making functional prototypes via selective laser sintering. They were PA_2200, PA_2210_FR, PA_3200_GF, PA_Alumide, and PA_640_GSL. These powders were manufactured by Electro Optical Systems (EOS, Germany), except for PA_640_GSL, which was manufactured by Advanced Laser Materials (ALM, USA) [54,55]. Some powders were not used as 100% new materials but were commonly processed with the same type of material already used as recommended by the supplier. The so-called refreshment factor testifies to this effect. Samples were printed with the recommended refresh factor, which is listed in Table 1.

### 2.2. Analysis of Powders

The thermal decomposition of the powders was determined by a combination of differential thermal analysis (DTA) and thermogravimetric analysis (TGA) using an STA 504 thermal analyzer (TA Instruments, New Castle, DE, USA). This method was used for real-time measurements of the weight loss of the examined materials as a function of temperature. Measurements were performed in a nitrogen (N_2_) atmosphere (nitrogen flow was 5 L·hod^−1^) at a heating rate of 20 °C·min^−1^ in the temperature range 30–550 °C. The weight of the tested sample was around 10 mg. T_max_ was evaluated from the derivation of the temperature loss curve as a function of temperature.

The range of size powder distribution was observed using a Keyence VHX-6000 confocal microscope (Keyence, Mechelen, Belgium) with 50× magnification and functioning in Grain Size Analysis mode.

### 2.3. Preparation of 3D Samples

3D printing was performed with an EOS P 396 LPBF printer, manufactured by Electro Optical Systems (EOS, Krailling, Germany). The technical parameters of the printer are listed in Table 2. Models have been created and edited using SOLIDWORKS 2020 (Dassault Systèmes, Vélizy-Villacoublay, France, version 2020) and exported in Standard Triangle Language “STL” format. Digital models were sliced using EOS RP-Tools 6.210 (EOS GmbH, Germany, Munich, version 2016) and were converted for 3D printers using PSW 3.8 EOS (EOS GmbH, Munich, Germany, version 2016). The relative humidity during the processing of the powders was 45%.

**Figure 1:**
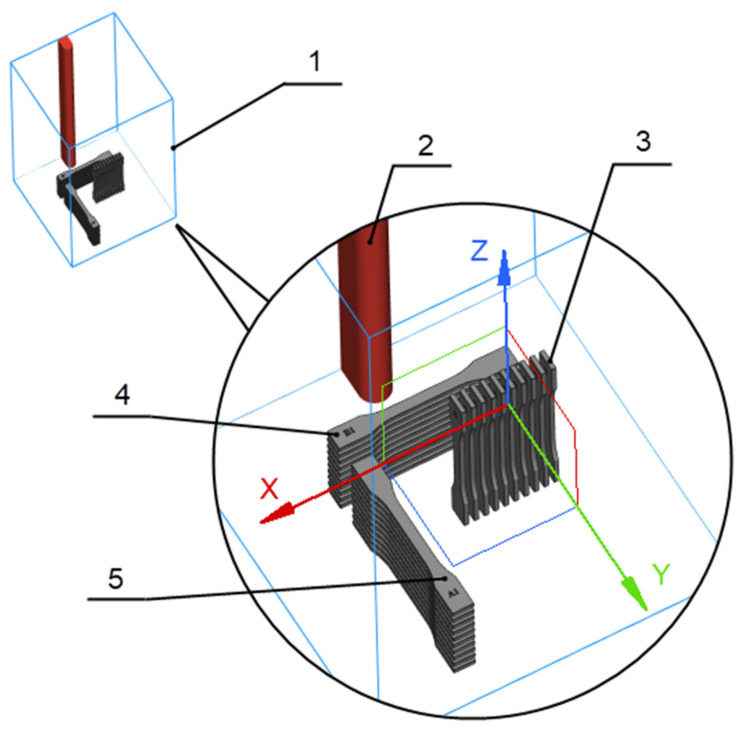
Part orientation and position in the 3D printer chamber; 1—Build Chamber EOS P396, 2—No Build Zone (red line), 3—Samples G1 (10 pcs), 4—Samples B1 (10 pcs), 5—Samples A1 (10 pcs).

The samples were printed as dog bones with dimensions specified in CSN EN ISO 527-2 [59]. The samples’ orientation and position in the build chamber are presented in Figure 1. Ten replicas of the samples were printed from each type. The 3D printed parameters such as layer thickness, temperature, beam offset, and scaling are listed in Table 3. Some parameters (such as temperature in the process chamber, beam offset) differ for individual types of materials. This is due to the specific composition of the material, the content of the specific additive, the refreshing factor for mixtures used, the melting point of the material, etc.

### 2.4. Analysis of 3D Samples

A Keyence VHX-6000 confocal microscope (Keyence, Mechelen, Belgium) with a VHX-S600E free-angle observation system (Z-motorized) was used for characterization of samples in terms of surface quality, color—their red, green, blue (RGB) system—and roughness. Surface roughness (Ra: arithmetical mean roughness value and Rz: mean roughness depth) was determined using ISO 25-178 [60]. Cross-sections of the samples were assessed to illustrate the distribution of additives, and morphology was obtained with magnification 2000x. The figures were analyzed using a free converter from the RGB system to CIE L*a*b* system www.easyrgb.com (accessed on 20 February 2022) to get the values of L*, a* and b*. The coordinates represent the lightness of the color (from L* = 0 black to 100 diffuse white), its position between red and green (a*, negative values indicate green, positive values indicate red) and its position between yellow and blue (b*, negative values indicate blue, positive values indicate yellow). The color change (ΔE*) was calculated according to the equation (Equation (1)):

ΔE* = √((ΔL*)^2^ + (Δa*)^2^ + (Δb*)^2^) (1)

To determine the wettability of the samples, the size of the contact angle was measured. Wettability is important as it can affect the quality of the surface and, potentially, the mechanical properties. The measurements were performed using a See System instrument (Advex Instrument, Brno, Czech Republic), where five measurements were performed for each sample and the mean and standard deviation were calculated. Water was chosen as the liquid, the size of the drop was 10 µL, and the reading of the size of the contact angle was taken after 10 s. The measurement was performed at room temperature (RT, 23 ± 2 °C).

Water absorption was determined using an Analyzer MB 23 (Ohaus Corporation, Parsippany, NJ, USA), which uses infrared to heat the samples up to 160 °C. The Analyzer MB 23 measures weight and humidity in samples from 0.5 to 20 g with 10 g/1% accuracy.

The dimensional analysis of the samples was performed using a caliper. The thickness and width of every sample were measured at three points from the center of the sample. Averages and standard deviations were calculated, the results are expressed as differences from the drawing documentation in percentage. A Citizen CY 104 analytical balance (Citizen Scales, Aczet Pvt. Ltd., Mumbai, India), which has a capacity of 120 g with an accuracy of 0.1 mg, was used to check the weights of the printed samples.

A universal mechanical device (Instron 3345, Instron, Norwood, MA, USA) with a maximum capacity load of 5 kN was used to test the tensile properties of the samples. The tensile test was undertaken at a speed of 1 mm·min^−1^. The test was performed according to ISO 527-2 [59].

**Figure 2:**
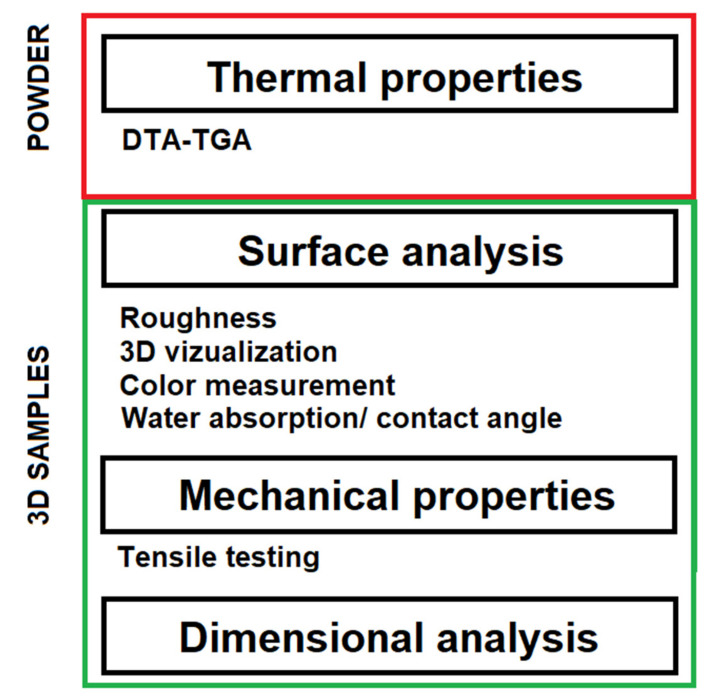
Scheme of material testing.

A summary of the analysis carried out for the powder and 3D samples can be seen in Figure 2.

## 3. Results

### 3.1. Analysis of Powders

DTA-TGA characterized powder samples of all the tested materials to determine their thermal properties. This ensures that the correct parameters for 3D printing were used. The chamber temperature was set using the measured temperatures of the powders, where the chamber temperature was higher in comparison with the melting temperature for the good sintering of powders [61]. The results of the thermal analysis can be found in Table 3. The samples were characterized by melting point, maximum temperature, and additive content. The PA_2200 sample does not contain additives and its melting point was determined to be 176.5 °C, which correlates with the literature on polyamide 12 [62]. The PA_640_GSL sample had a melting point of 184.2 °C and an 18.68 wt.% of the hollow glass and carbon fibers mix. Therefore, the additive content has an effect not only on physical properties but also on mechanical [63,64] and technological properties. PA_2210_FR powder contained 8.13 wt.% of halogen-free flame retardant and the melting point was determined at 186.9 °C. PA_3200_GF contained 38.35 wt.% of glass fibers, a melting temperature of 188.5 °C, and the last sample PA_Alumide contained 51.41 wt.% of alumina powder and a 184.7 °C melting temperature.

**Figure 3:**
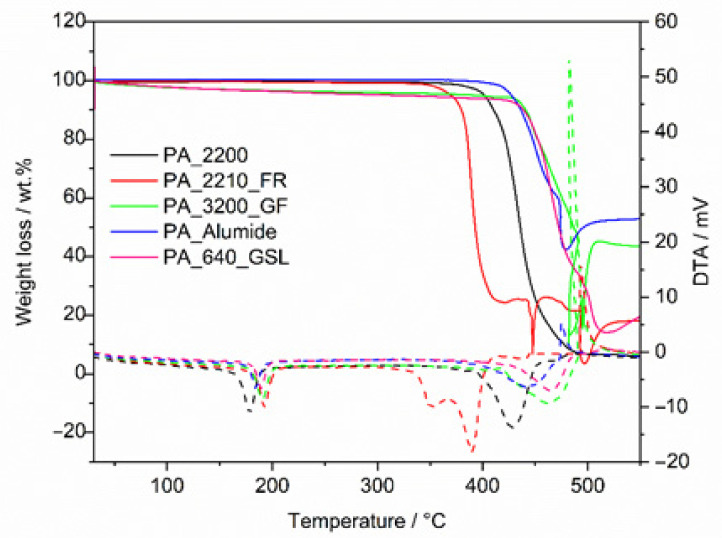
DTA-TGA graphs for all samples, where solid lines belong to weight loss, and dashed lines belong to DTA.

The results show the effect of additives on the specified melting temperature; after adding additives, it increased by about 8 °C. Furthermore, the samples were also characterized in terms of T_max_. Most of the samples containing additives showed higher heat resistance when compared to pure PA_2200. Only the sample PA_2210_FR, which contained flame retardant, showed a lower T_max_ than the PA_2200. The courses of thermal behavior can be seen in Figure 3. The content of additives in the polymer matrix has a great influence on T_max_ (it is a thermal characteristic about the temperature at which the sample loses its maximum weight), and since it is different, it is difficult to draw closer conclusions. A lot depends on the type of aid related to the possibility of applying different maximum amounts. However, from the application’s point of view, it is certainly important to know the processed materials in terms of their thermal properties. Another important characteristic in terms of thermal parameters is determining the so-called sintering window, which is the temperature interval between melting and crystallization onset points. As the DTA-TGA technique is not as sensitive as a differential scanning calorimetry (DSC), in our case, it was not possible to determine this important parameter in terms of LPBF technology. This parameter can be used, for example, to monitor the reuse of materials [38].

An important process parameter is the size of the particles used. The larger the particles, the higher the likelihood of inhomogeneity in the 3D product. This can increase the potential for crack propagation and lower the mechanical and technological properties. The results of the size distribution analysis can be found in Table 3. Particle size, shape, and distribution of powders are known to affect the surface roughness and porosity of components printed using LPBF technologies [65]. In addition, the smaller the particle size, the larger the specific surface area of the particles was observed [66].

**Figure 4:**
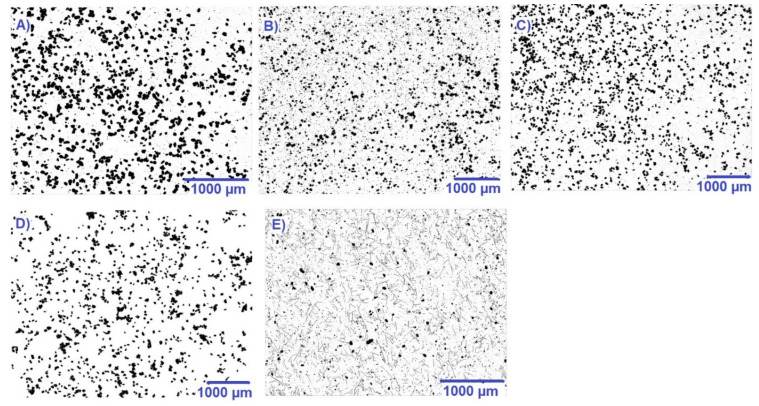
Images from powders observed by confocal microscopy Keyence VHX 6000, (**A**)—PA_2200, (**B**)—PA_2210_FR, (**C**)—PA_3200_GF, (**D**)—PA_Alumide, (**E**)—PA_640_GSL.

In terms of the average particle size of the powders used, the results are shown in Table 4, and the shape of powders can be seen in Figure 4. The average particle diameter size for the tested sample is comparable from this point of view. The results for PA_2200 are correlated with the particle diameter results reported in the study [67]. In general, the average particle size of polymer-based materials for LPBF processing is around 50–90 μm [68]. The roughness will not be affected by the size of the particles but by their shape, where there is a prediction of a stronger influence of fiber-shaped particles (glass fiber [69], carbon fiber [70]) than those in the form of spherical particles [71] (aluminum powder, flame retardant additive) on the surface properties of the 3D product.

### 3.2. Analysis of 3D Samples

**Figure 5:**
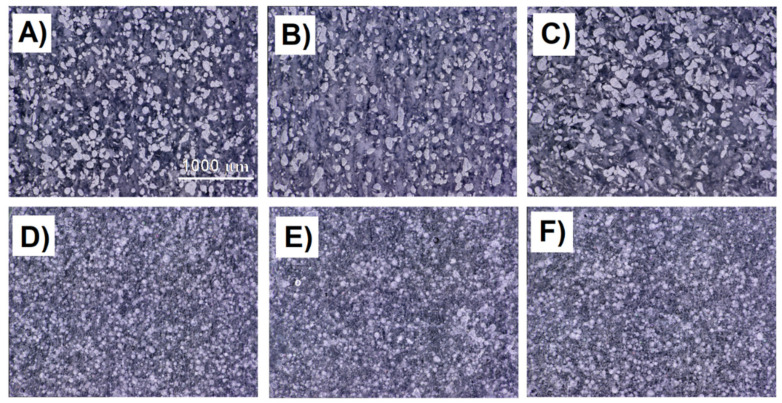
Cross-section of samples observed by confocal microscopy Keyence VHX 6000: (**A**) PA_Alumide, A1; (**B**) PA_Alumide, B1; (**C**) PA_Alumide, G1; (**D**) PA_640_GSL, A1; (**E**) PA_640_GSL, B1; (**F**) PA_640_GSL, G1.

The samples of PA_640_GSL and PA_Alumide were observed using a digital optical microscope as they were colored samples, and they had better visibility under the digital optical microscope. The samples of PA_2200, PA_2210_FR, and PA_3200_GF could not be observed due to white staining and poor visibility. The photo of PA_2200 was presented in the paper [36]. However, the same distribution was assumed for all materials. Figure 5 shows the distribution of the additives for the three print orientations. Figure 5A–C show the distribution of additive particles in a sample prepared from PA_Alumide. The distribution appears uniform in all three. However, the A1 sample appears to be the most homogeneous. For the samples prepared from PA_640_GSL, the samples showed a comparably homogeneous distribution (Figure 5D–F). From these observations, it could be argued that the homogeneous distribution of different types of additives was determined. The observation does not show the effect of the sample orientation during printing on the particle distributions in the printed samples with regard to the type of additive used.

Păcurar et al. [37] was engaged in the study of PA2200 material processed using LPBF technology, where the influence of the fracture surface related to the production parameters displayed using a scanning electron microscope (SEM) was demonstrated. The influence of the energy density on the porosity of a 3D product made of PA_2200 was also studied when the structure was monitored by SEM. The samples built with medium energy density exhibited lower porosity, while at low and high energies, the porosity was higher [72].

The samples were also characterized in terms of color measurement according to the CIEL*a*b* system. CIEL*a*b* is currently the most popular way of describing color and is the basis of modern color management systems [73]. The difference between two colors in a CIEL*a*b* space is the usual Euclidean distance Δ*E* between two points in three-dimensional space. It is assumed that the standard observer notices a color difference as follows: 0 < Δ*E* < 1—doesn’t notice the difference, 1 < Δ*E* < 2—only an experienced observer notices the difference, 2 < Δ*E* < 3.5—an inexperienced observer also notices the difference, 3.5 < Δ*E* < 5—notices a clear color difference, 5 < Δ*E*—the observer has the impression of two different colors. It can be seen from the color measurement results (Table 5) that there is a slight scatter when comparing samples printed in different directions for the same material. However, for each powder, the variance of the results is within the maximum measured deviation. The results ΔE* show how much the samples differ when compared to the reference (in our case with PA_2200). The higher the number, the more obvious the change.

ΔE* values indicate that the highest difference compared to the reference is shown by samples with aluminum content, which were values around 10.5. The results of the characteristics for determining the color are important when choosing materials for a specific application with regard to the requirements of, for example, the customer.

From the theoretical assumption, the higher the surface roughness, the easier that cracks can propagate under mechanical stress following a fracture [74]. Therefore, the surface roughness of the prepared 3D samples was determined. Results for all samples can be seen in Table 6. According to the theoretical assumption, the highest roughness values were measured in all the G1 samples. Samples printed in the A1 and B1 orientations were comparable in terms of measurements. The results show the same trend with respect to the direction of printing, which corresponds to the results presented in [75]. Roughness values are comparable for a 45 and 90° measuring angle; however, a 0° measuring angle gave a high surface roughness. Roughness significantly influences the contact angle measurement of a flat surface for various processes [76].

A disadvantage of using polyamides in 3D printing is that they have a tendency to absorb water [77]. For this reason, the contact angles of the printed samples were determined, which enables the materials to be classified as hydrophobic or hydrophilic. The results of the contact angle measurement can be found in Table 5. A material is classified as hydrophilic when the contact angle is smaller than 90°; if it is greater than 90°, it is classified as hydrophobic [78]. The results show that the print orientation has an effect on the contact angle. In general, G1 samples showed higher hydrophobicity when compared to the A1 and B1 samples. The most hydrophobic sample was the pure PA_2200 sample. Hydrophobicity decreased from print type G1 through B1 to A1 for all materials, except for the PA_640_GSL sample. This may be because the material contained large particles of additive, as can be seen in the microscope image (Figure 4). A similar trend was also observed in the study by Modi et al. for 3D technology fused filament fabrication (FFF), where samples prepared with print orientation 90° showed the highest values of contact angles [79].

From the point of view of the applicability of thermoplastics, polyamides are among the best structural polymeric materials which provide excellent mechanical properties. As these are water-absorbing materials, there is a drive to optimize their processing method as much as possible. The results, presented in Table 5, indicate that with the addition of additives, absorbency increases. The highest absorbency value was found in the PA_2210_FR sample, which contains a flame retardant and the PA_640_GSL sample, which contains a combination of carbon and glass fibers with 1.08 wt.%. Research has shown that carbon fiber has high absorbency [80]. On the contrary, the sample of PA_3200_GF had a low value of absorbency, which indicates that glass fibers themselves have a low absorbency value. The results were compared with the pure PA 12 sample.

In terms of mechanical testing, the worst results were expected for the G1 print orientation, while the A1 and B1 print orientation results were expected to be comparable. From the results presented in Table 7, it can be seen that the results were as predicted. The highest maximum load was applied to the PA_2200 sample, and the tensile strength of the samples decreased with the addition of additives. Comparable values for the reference material PA12 (PA_2200) were measured in the tensile test, namely, the determination of Young’s modulus 1270 ± 71 MPa [13], and tensile strength was about 43 MPa in orientations A1 and B1; results are comparable with a previous study [81]. On the contrary, the toughness of the four samples containing additives was higher, which can be seen in the higher Young’s modulus values. Since the parameters supplied by the manufacturer for each material were used, the aim was not to monitor the effect of laser power, scanning speed, or scanning pitch. It was observed [82] that the mentioned parameters affected tensile strength results. It was found for PA_2200 [82] that the best parameter combination was laser power 40 W, scanning speed 3000 mm·s^−1^, scanning pitch 0.4 mm, and tensile strength up to 46.42 MPa, from the perspective of tensile strength, processing cost, and processing cycle.

It is important to determine the ability of each material to produce a sample that conforms to the dimensions of the drawing (Figure 2). The dimensional analysis results are presented in Table 8. Positive values correspond to measured dimensions, which are above the drawing dimensions, while negative values indicate the opposite.

When comparing samples according to the print orientation, it was found that the samples printed with the A1 and B1 orientation are relatively comparable to G1. However, with the PA_2200 sample, the difference in thickness and width dimensions for the A1 and B1 print orientation was comparable, but G1 shows a smaller increase in size. With the addition of additives, a more significant effect on the dimensions of the samples was expected. The highest increase in dimensions can be seen in the PA_640_GSL sample, which contained a combination of glass fiber and carbon fibers. There was an increase in thickness of over 10% for the A1 and B1 orientations and an increase in width of over 2% for all three orientations. On the contrary, the dimensions of the PA_Alumide sample were smaller. A similar analysis was performed in [81] where there were similar trends for the results of pure PA 12 (PA_2200), there was also an increase in thickness, as well as a small change in width.

The weights of the tested samples were measured, as this is an important parameter in terms of practice. The average weights of the samples are presented in Table 8. As expected, the PA_Alumide sample with the aluminum-based additive has the highest weight, of 12.93 g ± 0.15 g.

## 4. Conclusions

This study systematically compared the thermal, surface, and mechanical properties of printed samples of pure polyamide 12, PA_2200, and materials containing various additives, PA_2210_FR, PA_3200_GF, PA_Alumide, and PA_640_GSL. These materials are commonly used in the mobility, aerospace, defense, and space sectors.

The research was conducted using dog-bone-shaped samples for three printing orientations in a chamber with a layer thickness of 0.12 mm. The default printing parameters recommended by the 3D printer manufacturer, EOS GmbH, and the powder material manufacturers were used for production. The study results show that the additive content affects not only the physical properties but also mechanical and technological properties. Advantages of this technique (LPBF) in comparison with the other techniques used to process polymeric materials are that it is self-supporting and allows reuse of the powder [83] The conclusions can be drawn as follows.

Temperature properties: From the temperature properties tested, it can be said that the starting material PA_2200 has the lowest melting temperature of 176.5 °C. The remaining materials have a melting point between 184.2 (PA_Alumide) and 186.9 °C (PA_2210_FR). The temperature curves also show that the samples absorb different amounts of moisture. The PA_640_GSL material, which contains glass and carbon fibers, and the PA_3200_GF material, which contains glass particles, showed the highest water absorption.

The particle size of powdered material: In terms of the particle diameter of the powders used, the range of particle size diameter is comparable between the tested powder. After viewing cross-sections of the samples under an optical microscope, it appeared that the larger the particles, the greater the possibility of non-homogeneity in the 3D product. Non-homogeneity increases the potential for crack propagation and reduces mechanical and technological properties.

Surface roughness parameters: The highest roughness values were measured for the G1 samples (printing of the samples in the direction of the *z*-axis), which confirmed the theoretical assumption. The worst surface quality was reported in the G1 samples made from PA_2200 (Rz = 120.7 µm) and PA_Alumide (Rz = 120.2 µm). The best surface quality was achieved on the G1 sample for the PA_640_GSL material (Rz = 85.133 µm). The A1 and B1 samples were comparable in terms of measurements. It was also found that the volume of additives in the powder affects the surface quality.

Mechanical properties: The worst results were expected for the G1 samples and comparable results were expected for the A1 and B1 samples. From the results in Table 6, this hypothesis was confirmed. Furthermore, it appears that the highest tensile strength values were measured for the PA_2200 sample (A1: 44.4 MPa; B1: 43.2 MPa; G1: 39.5 MPa). With the addition of different types of additives, the tensile strength limits decreased. The lowest tensile strength was observed for the PA_640_GSL material (G1: 26.5 MPa).

The addition of additives improved the toughness of the samples, as seen in the higher values of Young’s modulus (for example, PA_Alumide; G1: 2 570.92 MPa).

This study demonstrates the importance of additives in pure PA 12 powder and their advantages and disadvantages with respect to mechanical, physical, and technological properties. Designers can select a suitable material and these results can assist them with their 3D model design. Reasons for this may include the inaccuracy of production in the design process, due to the thermal process and shrinkage, and allows a choice of the appropriate orientation of the model in 3D printing, while considering the required mechanical properties and surface roughness parameters. For materials prone to water absorption, designers may select appropriate post-process surface treatment (e.g., varnishing).

For most applications, reducing weight is very important. Introducing additives makes it possible to design models with the required mechanical properties whilst reducing the final product’s weight for samples PA_640_GSL. Considering the development trends in additive manufacturing, the development of new multi-material properties can be expected in the near future. The application potential of such materials can be expected for smart, soft, and flexible composite models.

## Figures and Tables

**Table 1:** Powder refreshing [56].

Material	New Powder/wt.%	Old Powder/wt.%
PA_2200	50	50
PA_2210_FR	100	-
PA_3200_GF	70	30
PA_Alumide	100	-
PA_640_GSL	70	30

**Table 2:** Technical data of 3D printers [57,58].

Technical Data	EOS P 396
Construction volume (dimensions)	340 × 340 × 620 mm^3^
Building rate	up to 3 L·h^−1^ *
Laser type and power	CO_2_, 70 W
Precision optics	F-theta lens, high-speed scanner
Scan speed	up to 6 m·s^−1^

* with a packing density of 5%.

**Table 3:** Printing parameters.

Polyamide Powders	Layer Thickness/mm	Temperatures/°C		Beam Offset/mm	Material Dependent Scaling/%			
		Process Chamber	Removal Chamber		X	Y	Z (0)	Z (600)
PA_2200	0.12	171	130	0.35	3.20	3.20	2.55	1.40
PA_2210_FR		178		0.33	2.40	2.40	1.70	1.20
PA_3200_GF		180		0.31	3.40	3.40	2.20	1.60
PA_Alumide		178		0.33	2.00	2.20	1.50	1.10
PA_640_GSL		175		0.36	1.15	2.15	2.80	1.60

**Table 4:** Results for DTA-TGA measurement and size distribution.

Sample	Melting Point/°C	T_max_/°C	Additive Content/wt.%	AVG Size of Particle/µm
PA_2200	176.5	431.7	-	67.81
PA_2210_FR	186.9	383.7	11.38	53.94
PA_3200_GF	188.5	443.9	42.10	88.93
PA_Alumide	184.7	449.8	54.71	56.81
PA_640_GSL	184.2	460.2	22.84	26.81

**Table 5:** Results for color measurement, STD up to 5%.

Sample	Print Orientation	L*	a*	b*	ΔE*
PA_2200	A1	71.11	6.63	−24.26	*
	B1	71.73	6.26	−24.89	*
	G1	69.75	6.50	−25.49	*
PA_2210_FR	A1	69.16	6.65	−24.19	1.95
	B1	69.78	6.99	−23.95	2.28
	G1	69.57	7.02	−26.67	1.30
PA_3200_GF	A1	65.53	5.26	−20.32	6.97
	B1	65.56	6.93	−25.69	6.26
	G1	65.05	11.03	−24.19	6.66
PA_Alumide	A1	61.36	4.21	−19.99	10.92
	B1	62.96	5.18	−21.72	11.04
	G1	62.28	6.55	−20.13	9.19
PA_640_GSL	A1	67.64	8.63	−25.95	4.35
	B1	67.43	8.87	−26.09	5.17
	G1	67.65	6.66	−21.60	4.42

* do not calculate, the reference value.

**Table 6:** Results for roughness and contact angle measurement.

Sample	Print Orientation	Mean Roughness Value, Ra/µm	Mean Roughness Depth, Rz/µm	Contact Angle/°	Water Absorption/wt.%
PA_2200	A1	12.1 ± 0.03	74.7 ± 0.02	89.4 ± 6.6	0.62 ± 0.17
	B1	12.8 ± 0.01	72.5 ± 0.03	96.4 ± 3.4	
	G1	25.3 ± 0.03	120.8 ± 0.05	110.5 ± 3.3	
PA_2210_FR	A1	8.6 ± 0.01	87.3 ± 0.03	89.9 ± 4.8	1.00 ± 0.21
	B1	6.6 ± 0.01	64.4 ± 0.02	97.7 ± 2.3	
	G1	18.4 ± 0.03	91.3 ± 0.04	98.4 ± 6.2	
PA_3200_GF	A1	9.8 ± 0.02	49.8 ± 0.03	86.2 ± 5.3	0.59 ± 0.05
	B1	12.4 ± 0.02	73.4 ± 0.02	94.5 ± 2.9	
	G1	18.4 ± 0.03	91.3 ± 0.03	100.3 ± 6.2	
PA_Alumide	A1	9.1 ± 0.01	50.2 ± 0.03	85.9 ± 4.9	0.61 ± 0.33
	B1	9.9 ± 0.01	51.8 ± 0.01	86.6 ± 4.7	
	G1	25.4 ± 0.03	120.2 ± 0.04	92.3 ± 5.6	
PA_640_GSL	A1	9.1 ± 0.01	49.5 ± 0.03	90.7 ± 9.9	1.08 ± 0.08
	B1	13.1 ± 0.02	54.9 ± 0.03	96.6 ± 4.9	
	G1	15.1 ± 0.03	85.1 ± 0.02	85.7 ± 7.6	

**Table 7:** Tensile properties.

Sample	Print Orientation	Tensile Strength/MPa	Young’s Modulus/MPa
PA_2200	A1	44.4 ± 0.9	1387.4 ± 51
	B1	43.2 ± 0.4	1290.1 ± 26
	G1	39.5 ± 2.4	1339.2 ± 23
PA_2210_FR	A1	34.9 ± 0.2	2024.9 ± 30
	B1	35.3 ± 0.4	1986.4 ± 60
	G1	31.4 ± 0.2	1867.4 ± 44
PA_3200_GF	A1	31.9 ± 0.7	2362.8 ± 74
	B1	32.8 ± 0.5	2211.5 ± 97
	G1	32.2 ± 1.0	2180.3 ± 46
PA_Alumide	A1	33.9 ± 1.1	2660.1 ± 127
	B1	33.8 ± 0.4	2492.9 ± 127
	G1	32.9 ± 0.6	2570.9 ± 31
PA_640_GSL	A1	30.8 ± 0.9	1996.3 ± 43
	B1	33.8 ± 1.2	2306.5 ± 37
	G1	26.5 ± 0.4	1807.4 ± 19

**Table 8:** The dimension analysis of the printed samples.

Sample	Print Orientation	Width Difference/%	Thickness Difference/%	Weight/g
PA_2200	A1	0.60 ± 0.02	5.43 ± 0.02	9.68 ± 0.11
	B1	0.91 ± 0.02	5.98 ± 0.02	
	G1	0.47 ± 0.06	2.73 ± 0.07	
PA_2210_FR	A1	−0.15 ± 0.01	−2.73 ± 0.02	10.72 ± 0.05
	B1	0.18 ± 0.02	0.13 ± 0.02	
	G1	1.65 ± 0.02	5.98 ± 0.03	
PA_3200_GF	A1	−0.72 ± 0.02	−4.27 ± 0.03	12.19 ± 0.07
	B1	−0.31 ± 0.02	−1.85 ± 0.01	
	G1	0.66 ± 0.02	4.10 ± 0.02	
PA_Alumide	A1	−4.20 ± 0.40	−4.12 ± 0.02	12.93 ± 0.15
	B1	−1.83 ± 0.02	−2.40 ± 0.04	
	G1	−0.71 ± 0.03	−0.67 ± 0.02	
PA_640_GSL	A1	2.39 ± 0.05	10.87 ± 0.06	8.96 ± 0.08
	B1	2.77 ± 0.11	14.17 ± 0.08	
	G1	2.23 ± 0.04	3.52 ± 0.03	

## Data Availability

The data presented in this study are available upon request from the corresponding author.
